# FPGA Implementation of AI-Based Inverter IGBT Open Circuit Fault Diagnosis of Induction Motor Drives

**DOI:** 10.3390/mi13050663

**Published:** 2022-04-23

**Authors:** Nagalingam Rajeswaran, Rajesh Thangaraj, Lucian Mihet-Popa, Kesava Vamsi Krishna Vajjala, Özen Özer

**Affiliations:** 1 Electrical and Electronics Engineering, Malla Reddy Engineering College, Secunderabad 500100, India; rajesh.t@mrec.ac.in; 2 Faculty of Information Technology, Engineering, and Economics, Oestfold University College, 1757 Halden, Norway; 3 Department of Physics, Malla Reddy Engineering College, Secunderabad 500100, India; media@mrec.ac.in; 4 Department of Mathematics, Faculty of Science and Arts, Kırklareli University, Kırklareli 39100, Turkey; ozenozer39@gmail.com

**Keywords:** condition monitoring, Induction Motor Drive, fault diagnosis, FPGA, Back Propagation Neural Network, Discrete Wavelet Transforms

## Abstract

In modern industrial manufacturing processes, induction motors are broadly utilized as industrial drives. Online condition monitoring and diagnosis of faults that occur inside and/or outside of the Induction Motor Drive (IMD) system make the motor highly reliable, helping to avoid unscheduled downtimes, which cause more revenue loss and disruption of production. This can be achieved only when the irregularities produced because of the faults are sensed at the moment they occur and diagnosed quickly so that suitable actions to protect the equipment can be taken. This requires intelligent control with a high-performance scheme. Hence, a Field Programmable Gate Array (FPGA) based on neuro-genetic implementation with a Back Propagation Neural network (BPN) is suggested in this article to diagnose the fault more efficiently and almost instantly. It is reported that the classification of the neural network will provide the output within 2 µs although the clone procedure with microcontroller requires 7 ms. This intelligent control with a high-performance technique is applied to the IMD fed by a Voltage Source Inverter (VSI) to diagnose the fault. The proposed approach was simulated and experimentally validated.

## 1. Introduction

Industrial induction motors are highly reliable and easy to operate; hence, they are extensively used as industrial drives [1,2]. They work under harsh and severe conditions, and as a result, they are subject to both internal and exterior faults and breakdowns [3]. These faults must be sensed at the earliest stage; otherwise, catastrophic failure of the machine may result in disruptions to production [4,5]. It was this need that necessitated online supervision and fault analysis design to be integrated with the drive system [6,7].

According to conventional wisdom, the maintenance of Induction Motor Drive (IMD) happens at a certain interval. However, the performance of IMD may decline at irregular intervals as a result of environmental and operational factors. As a result, online monitoring of instant messaging is required to increase efficiency. In new evolving methodologies, predictive maintenance via condition monitoring (CM) is a critical component, intending to project the maintenance schedule based on the state of the plant or process [8,9,10]. It is possible to improve the performance and efficiency of an IMD by using condition-based monitoring. Such monitoring also extends the life and productivity of the system and reduces internal and external damages. It has become vital to use CM and fault detection in IMDs to prevent unexpected failures and reduce unplanned downtime. There are many ways for condition monitoring of IM, including Acoustic Emission (AE) monitoring, vibration signature analysis, and Motor Current Signature Analysis (MCSA). However, these monitoring techniques are complicated and need costly sensors [10]. When a CM system is efficient, it is capable of delivering early warning and forecasting errors. The CM system obtains basic data information from the motor via the use of signal processing or data analysis methods as described before. Although the method does not need human interpretation, it does have a fundamental downside [11,12,13]. The automation of the fault detection and diagnosis process is a natural evolution in the development of CM technologies [14,15]. An intelligent system, such as artificial intelligence methods, Genetic Algorithms (GA), Fuzzy Logic (FL), Artificial Neural Networks (ANN), and expert systems, is required for the autonomous fault detection system [16]. In an industry-based comprehensive assessment of high voltage IMD failures, multiple types of classification were used to identify the causes of the failures [17]. These categories included protection system, machine size, age, number of poles, maintenance regime, and operating hours. Induction machines have been subjected to an investigation into the causes of both stator and bearing failures, which together account for about 75% of all failures [18].

To gather information regarding CM and diagnostic measures, a survey on IM drives for industrial applications was conducted [19]. The research focused on the challenges that are now being addressed and those that will be addressed in the future in the development of autonomous diagnostic methods. The LabVIEW platform was used to produce cutting-edge capabilities for online control of induction motors [20,21,22]. It has been determined that the use of stator current analysis-based demodulation methods is the most suited method for diagnosing bearing faults.

There are many noncontact CM approaches that may be used to diagnose inductor motor failures. Specifically, it was discovered that the park vector analysis and instantaneous power analysis procedures are the most effective methods for recognizing motor failure signals. The Support Vector Machine (SVM)-based algorithms have demonstrated that they provide improved results for the classification and fault diagnosis of a three-phase induction motor [23]. The Bearing Damage Index (BDI), which is based on the wavelet packet node energy coefficient analysis method, has been proposed not only to detect faults in bearings but also to detect the severity level of the fault [24]. The Bearing Damage Index (BDI) is based on the wavelet packet node energy coefficient analysis method. A review of the most current literature has been published on the automation of condition monitoring in IMD [25]. When it comes to directing maintenance for electrical machines, one of the factors that has been identified as a barrier is the cost-to-benefit ratio between capital and operating expenses [26,27,28].

In the last two decades or so, condition supervision and fault identification of IMD attracted the attention of many researchers who developed AI-based control schemes such as expert systems, fuzzy interference systems, neural network and neuro-fuzzy techniques. All these techniques when implemented in real time are computationally complex, time-consuming, and lacking in optimal switching strategies. Hence, a new method, neuro-genetic design and implementation of fault diagnosis of induction motors based on a FPGA are proposed in this article [29,30]. The measured signals are processed through DWT for feature extraction. These features are used to detect the type of fault that occurred in the system.

The remaining article is structured as follows. The proposed test system model is presented in Section 2. Section 3 details the proposed method, and Section 4 presents the experimental results. Lastly in Section 5 the conclusions are presented.

## 2. Proposed System Description

**Figure 1:**
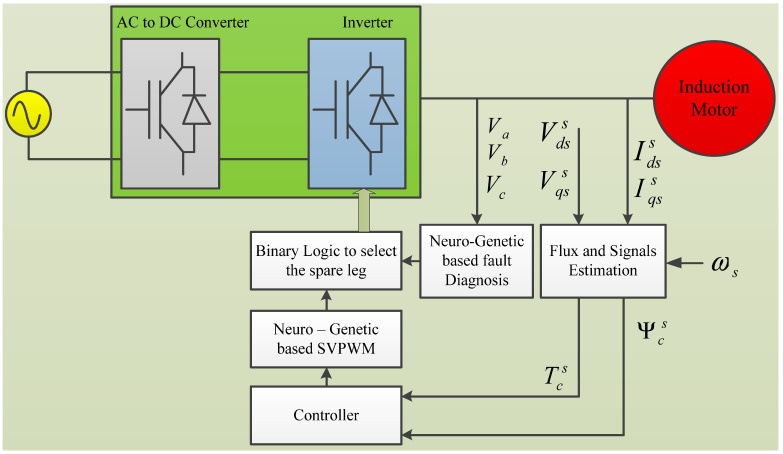
Schematic of Neuro-Genetic-based fault diagnosis drive system.

The schematic diagram of the IMD with a FPGA-based neuro-genetic implementation is shown in Figure 1. The proposed system consists of a power supply block having an AC to DC converter node and a DC to AC inverter node, a squirrel cage induction motor, a flux and signal estimation (Programmable Cascaded Low Pass Filter (PCLPF)) block, a neuro-genetic based fault diagnosis block, a controller block, a neuro-genetic-based Space Vector Pulse Width Modulation (SVPWM) block, and a binary block. The input signals corresponding to the induction motor’s terminal voltages and currents are transformed into output signals indicating torque and flux by the lux and signal estimation block. These signals are sent into the controller block, which creates input signals for the SVPWM block, which processes and generates suitable pulses for the binary logic block. The fault diagnosis block receives signals matching the Insulated Gate Bipolar Transistor (IGBT) inverter’s output voltages.

It processes them in the neural network to produce TRIAC signals for the modified structure of the IGBT inverter. These, together with the output signal of the SVPWM block, are input to the binary logic block, the output signals of which are utilized to transfer the jurisdiction from the faulty leg to the backup leg.

### 2.1. Reconfiguration of Inverter Topology

**Figure 2:**
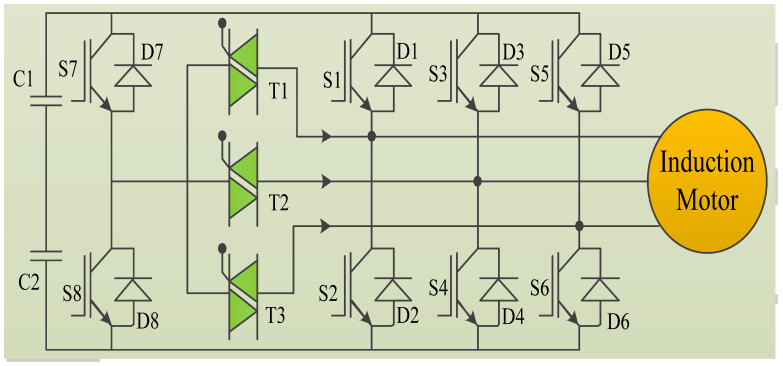
IGBT Inverter topology.

IGBT inverter topology is shown in Figure 2. The inverter structure has three legs, with every leg carrying two switches, **S1**, **S2**, **S3**, **S4**, and **S5**, **S6**, correspondingly. The fourth leg has another two switches, **S7** and **S8**. Three Triacs, **T1**, **T2** and **T3**, are utilized for configuring the inverter later fault existence and its elimination. If there is a misfiring in power switch **Sn**, the fault identification part finds this fault and separates the corresponding fault leg by disconnecting the gate signals to the switch **Sn**. The phase current ‘**i_sn_**’ is reduced to zero by the freewheeling diodes **Dn** in the faulted leg. Then the restructuring module fires **Tn** which interconnects ‘**n**’ (Leg) and ‘**o**’ (**C1** and **C2**).

### 2.2. Fault Diagnosis Based on a FPGA

To accurately diagnose the fault when it occurs, the trend or changes in the monitoring signals must be sensed unambiguously so that the same could be converted into binary code. These coded signals could be used to translate the fault category and its position. Then appropriate gate signals can be generated for suitable action on the system. To carry out the above processes, the control scheme should consist of facilities such as a signal or feature eradication, neural network regulation, fault identification, and gating signals. For the drive system under consideration, the fault diagnosis is performed as follows. The backfire in any one of the switches in a leg of the inverter can be recognized by an error in the corresponding leg voltage. The neural network is experienced with ordinary and extraordinary data for the inverter operation, and so outputs of the neural network are almost ‘0’ and ‘1’ as binary code. Then, corresponding to the error signal of the faulty leg voltage, binary code is generated and sent to the fault identification structure which senses and decodes the fault category and its location. Thereupon, the neural network selects the switches to isolate the faulty leg and bring in the spare leg so that the inverter regains its normal state as a three phase **VSI** to supply the IMD, making it fault tolerant. Suppose if the fault occurred in leg ‘**n**’, causing a deviation in the leg voltage ±ΔVno. Then, the leg voltage after fault occurrence can be given as Equation (1),

(1) $$V^{\prime }no=Vno\pm \Delta Vno$$

**Figure 3:**
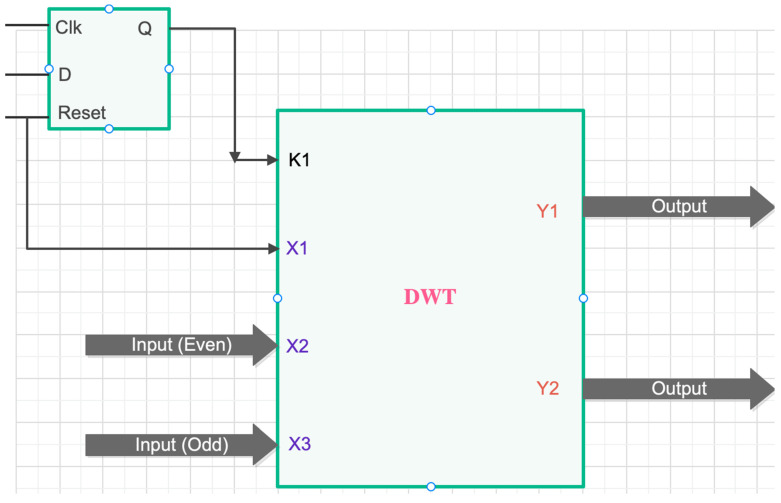
Schematic view of DWT.

This signal may not distinguish itself from Vno, and so a signal transformation technique is required to accurately diagnose the fault. The feature or signal extractor should be such that it provides adequate and significant details about the trend of the signal to enable the neural network to diagnose the fault type and its location with a high degree of accuracy. To achieve this, a feature extractor using a Discrete Wavelet Transform (DWT) technique is employed. The Register Transfer Language (RTL) schematic diagram of the DWT technique is shown in Figure 3. Discrete wavelet transformation is good in time resolution of high frequencies [11].

## 3. Neuro-Genetic Approach for Fault Classification

For accurate fault isolation, the signals (voltage, current, and speed) are transformed with the DWT technique for feature extraction [8]. After transformation, the output voltage variations are classified by using the neuro-genetic approach which continues to feedback the signals until desired (target) output is obtained representing the fault situation.

### Neuro-Genetic Architecture Design

**Figure 4:**
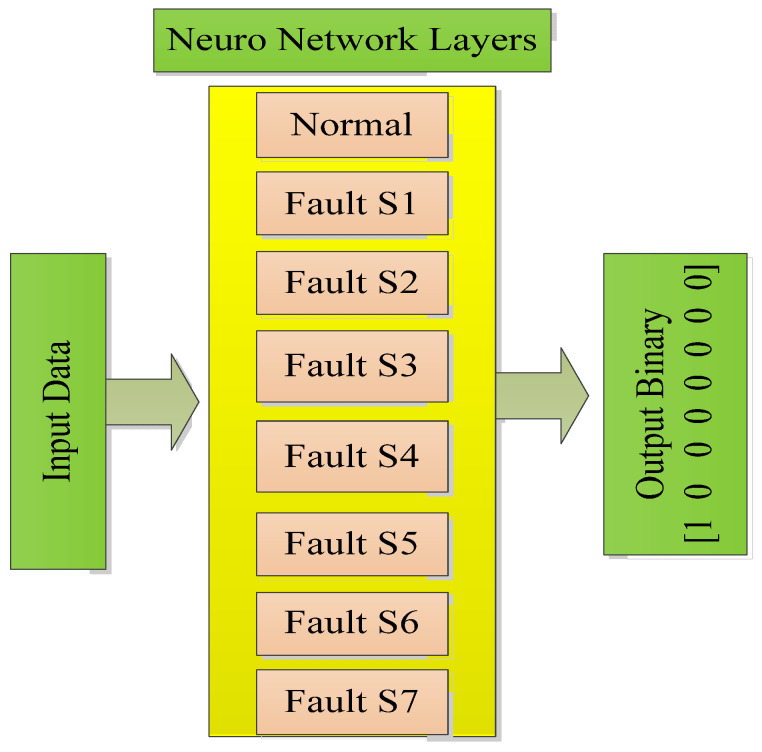
Development of BPN classification structure.

The structure of the BPN classification based on a FPGA is shown in Figure 4. For the given drive system fed by a **VSI**, there are seven states to represent the conditions, i.e., normal, fault on **S1**, fault on **S2**, fault on **S3**, fault on **S4**, fault on **S5**, fault on **S6**, and fault on **S7**. It requires a seven-layer neural structure. In addition, there are three hidden nodes and one yield node.

The neuro-genetic-SVPWM has double subnets. One is the voltage amplitude subnet with a 1-3-1 structure, and the other is the angle subnet with the architecture of 1-18-3 to produce a three-phase yield. The sigmoid activation function is used. Every structure is experienced with one set of normal data and four sets of faulted data.

**Figure 5:**
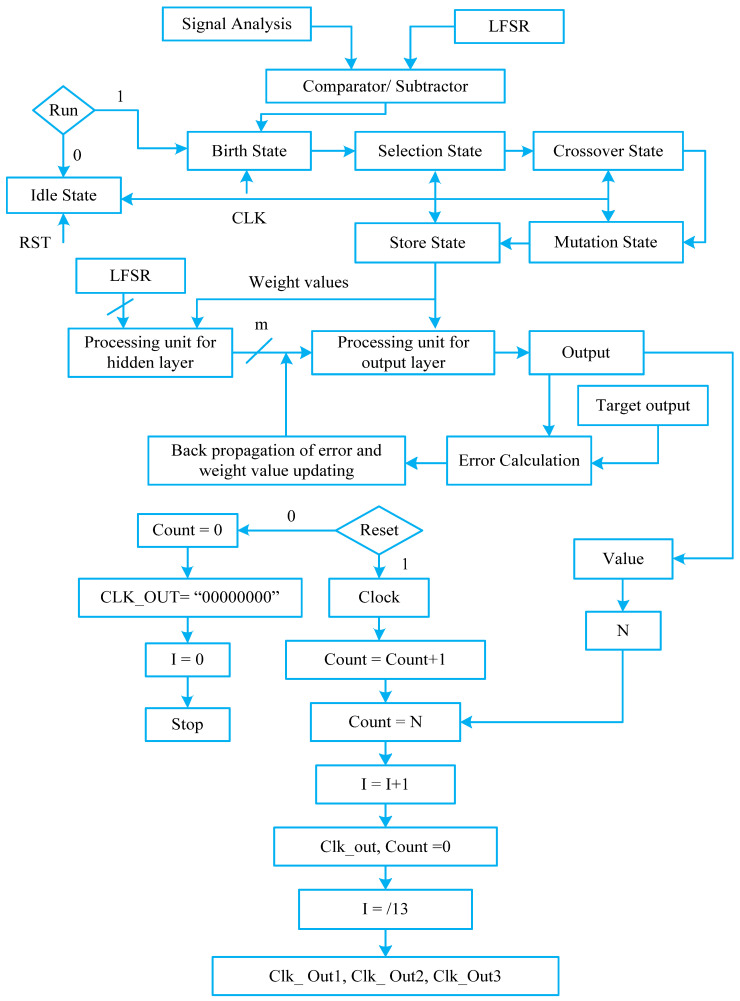
Flow chart of neuro-genetic design.

**Figure 6:**
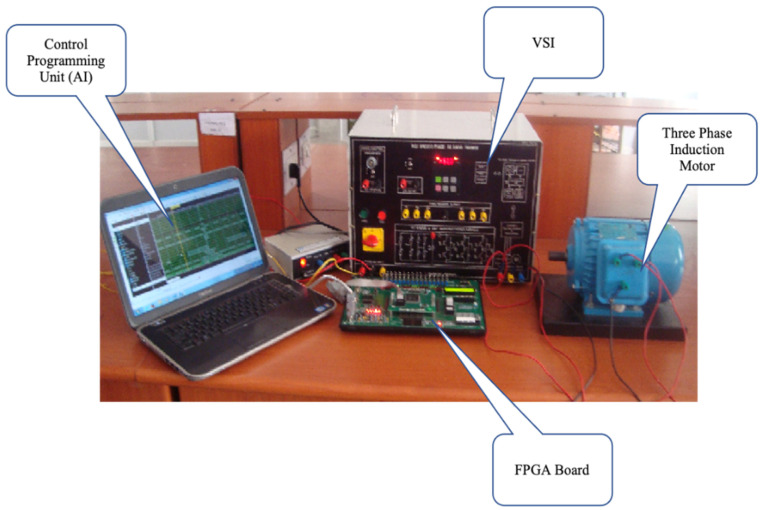
Neuro-genetic implementation based on a FPGA.

The use of GA helps achieve an optimized weight value for BPN to obtain the desired output for the fault situation. Thus, the neuro-genetic technique based on a FPGA when implemented functions in such a way that the control scheme is capable of fast processing to achieve fault diagnosis almost instantly [12,13]. Here, the mixed design of neural networks and genetic algorithms is developed and implemented in the FPGA process as given by the flow diagram in Figure 5. The idle, birth, selection, crossover, mutation, and store states are used in GA. Linear feedback shift register (LFSR) is used to produce arbitrary numbers [13,14]. A fitness value is designated to every part in the community depending on the discrepancy in the set and original output of the structure. The total number of Pins in the FPGA appliance is 208 and utilized pins in the suggested structure are only 49. The experimental setup presented in Figure 6 is verified on the xc3s500e-4-pq208 board (Xilinx, San Jose, CA, USA). The three-phase induction motor specifications are listed in Table 1.

## 4. Results and Discussion

The problem associated with ANN concerning weight optimization to train the network is adequately addressed using GA. The use of DWT as a feature extractor provides significant details in the pattern set to the neural network, enabling it to perform with a high degree of accuracy in fault diagnosis.

With successful configuration by a FPGA, the neuro-genetic-SVPWM processes the signals such that the variation of the neural network provides the yield with minimum and maximum time of 10.85 ns and 11.99 ns. It is reported that the classification of the neural network will provide the output within 2 µs although the clone procedure with a microcontroller requires 7 ms [17]. However, the neuro-genetic approach obtained the low and high period of yield as 10.857 ns (7.440 ns logic, 3.417 ns route, 68.5% for logic and 31.5% for route) and 11.99 ns (8.042 ns for logic, 3.952 ns for route, 67.1% for logic and 32.9% for route), respectively. The result simulations are performed by using integer numbers. The selected device power information is shown in Table 2. The prototype requirement of the proposed method device utilization summary is provided in Table 3. According to the defective and normal conditions, the output voltage waveform reveals how quickly the neuro-genetic process will produce the switching wave shape. A total of 173,524 kilobytes of RAM are used. The suggested design achieves the use of hardware and efficiency to minimize power consumption in different aspects. Table 4 shows the full clock reports and timing summary. The simulation timing and real-time clock utilization by VHDL is a hardware description language (HDL) software and is tabulated below in Table 5.

## 5. Conclusions

Fault diagnosis of IMD has been attempted for fault occurrence in VSI by using a neuro-genetic technique based on a FPGA. The neuro-genetic algorithm (BPN with GA) processes the error to diagnose the fault type and its location to transfer the switching from faulty leg to spare leg of the IGBT inverter, thereby making the system a fault tolerant IMD. The implementation of this technique is found to increase the speed of response for situational observation and fault identification, thereby enhancing the reliability of the drive system in modern industrial processes. With successful configuration by a FPGA, the neuro-genetic-SVPWM processes the signals such that the variation of the neural network provides the yield with minimum and maximum time of 10.85 ns and 11.99 ns. The proposed techniques can be extended in the near future with various machine-learning methods and switching response can be improved.

## Figures and Tables

**Table 1:** Induction motor specifications.

Parameter	Range
Speed	1390 rpm
Volts	415 V
Frequency	50 Hz
Power	0.75 kW
Pole	4

**Table 2:** Power summary.

Parameter	Power (W)	Voltage	Range	Icc (A)	Iccq (A)
Vccint	0.031	1.20	1.14 to 1.25	0.000	0.026
Vccaux	0.045	2.5		0.000	0.018
Vcco25	0.005	2.5		0.000	0.002

**Table 3:** Device utilization summary.

**Logic Utilization**	**Used**	**Available**	**Range**
Total number of slice registers	188	9312	2%
Number used as flip flops	105		
Number used as latches	83	2.5	
Number of 4 input LUTs	270	9312	2%
**Logic Distribution**	**Used**	**Available**	**Range**
Number of occupied slices	217	4656	4%
Number of slices containing only related logic	217	217	100%
Number of slices containing unrelated logic	0	217	0%
Total Number of 4 input LUTs	303	9312	3%
Number used as logic	270		
Number used as a route-through	33		
Number of bonded IOBs	81	159	51%
IOB latches	11		
Number of BUFGMUXs	3	24	12%
Number of M|ULT|I18X18SIOs	4	20	20%

**Table 4:** Clock Report.

Clock Net	Resource	Locked	Fanout	Net Skew (ns)	Max Delays (ns)
X4/y0_not001	BUFGMUX_X2Y10	No	12	0.011	0.142
Clk1_BUFGP	BUFGMUX_X2Y11	No	75	0.076	0.196
State_out1_1_OBUF	BUFGMUX_X1Y10	No	11	0.030	0.148
x3/ov4	Local		16	0.045	1.249
x3/ov1	Local		6	0.211	1.988
x3/ov3	Local		5	0.460	1.124
x3/ov2	Local		6	0.224	2.235

**Table 5:** Timing Summary.

Parameters	Frequency
Minimum period	10.857 ns
Maximum frequency	92.108 MHz
Minimum input arrival time before clock	20.18 ns
Maximum output required time after clock	11.99 ns
Maximum combinational path delay	8.610 ns
Total REAL time to Xst completion	11.00 s
Total CPU time to Xst completion	10.41 s
